# Cartilage-Specific Knockout of the Mechanosensory Ion Channel TRPV4 Decreases Age-Related Osteoarthritis

**DOI:** 10.1038/srep29053

**Published:** 2016-07-08

**Authors:** Christopher J. O’Conor, Sendhilnathan Ramalingam, Nicole A. Zelenski, Halei C. Benefield, Isaura Rigo, Dianne Little, Chia-Lung Wu, Di Chen, Wolfgang Liedtke, Amy L. McNulty, Farshid Guilak

**Affiliations:** 1 Department of Pathology & Immunology, Washington University in St. Louis, Missouri, 63110, USA; 2 UNC/NCSU Joint Department of Biomedical Engineering, UNC School of Medicine, Chapel Hill, NC 27599, USA; 3 Department of Orthopaedic Surgery, Duke University Medical Center, Durham NC 27710, USA; 4 Department of Orthopaedic Surgery, Washington University in St. Louis, Missouri, 63110, USA; 5 Shriners Hospitals for Children – St. Louis, St. Louis, Missouri 63110, USA; 6 Department of Biochemistry, Rush University, Chicago, IL, 60612, USA; 7 Department of Neurology and Neurobiology, Duke University Medical Center, Durham NC 27710, USA

## Abstract

Osteoarthritis (OA) is a progressive degenerative disease of articular cartilage and surrounding tissues, and is associated with both advanced age and joint injury. Biomechanical factors play a critical role in the onset and progression of OA, yet the mechanisms through which physiologic or pathologic mechanical signals are transduced into a cellular response are not well understood. Defining the role of mechanosensory pathways in cartilage during OA pathogenesis may yield novel strategies or targets for the treatment of OA. The transient receptor potential vanilloid 4 (TRPV4) ion channel transduces mechanical loading of articular cartilage via the generation of intracellular calcium ion transients. Using tissue-specific, inducible *Trpv4* gene-targeted mice, we demonstrate that loss of TRPV4-mediated cartilage mechanotransduction in adulthood reduces the severity of aging-associated OA. However, loss of chondrocyte TRPV4 did not prevent OA development following destabilization of the medial meniscus (DMM). These results highlight potentially distinct roles of TRPV4-mediated cartilage mechanotransduction in age-related and post-traumatic OA, and point to a novel disease-modifying strategy to therapeutically target the TRPV4-mediated mechanotransduction pathway for the treatment of aging-associated OA.

Osteoarthritis (OA) is a common cause of pain and disability in the general population, affecting over 27 million people in the United States^1^. Major risk factors for OA include age, obesity, and joint injury^2^. Non-surgical treatments can often provide a degree of symptomatic relief, but ultimately do not address the underlying disease process. Understanding the biologic and environmental factors that influence disease progression is critical to developing therapies that could slow or prevent OA pathogenesis^3^.

OA is characterized by an imbalance of anabolic and catabolic activities by resident articular chondrocytes, which promotes cartilage tissue degradation^4^,^5^,^6^. This loss of homeostatic balance is heavily influenced by biologic factors, such as aging^7^ and inflammation^8^, as well as biomechanical factors, such as altered joint kinematics related to obesity or following injury to the stabilizing menisci or ligaments of the knee^2^. In this regard, a better understanding of the pathways involved in biomechanically-mediated cartilage tissue homeostasis may provide important insights into the pathogenesis of OA, and furthermore, provide potential molecular or physical targets for new OA therapeutics.

Animal models that recapitulate the most clinically relevant mechanisms of OA include aging^9^, diet-induced obesity^10^, and joint destabilization^11^. Aging leads to a number of physiological changes within the joint, including alterations in the composition and mechanical properties of tissues^12^, as well as changes in the chondrocyte transcriptome^13^, both thought to contribute to the propensity of OA to develop during the aging process. Destabilization of the medial meniscus (DMM), a post-traumatic OA model involving the transection of the medial meniscotibial ligament, alters joint biomechanics and causes pathologic changes similar to those seen in human post-traumatic OA following ACL or meniscal tears^11^. While DMM-induced OA appears qualitatively similar to aging-associated OA^14^, the mechanisms of disease are dissimilar in many respects^13^,^15^, and few studies have examined both mechanisms in the same model^16^.

The transient receptor potential vanilloid 4 (TRPV4) membrane ion channel is part of a family of TRP channels that collectively play an important role in vertebrate physiology by acting as physiologic sensors^17^. As a calcium ion (Ca^2+^) preferred channel, TRPV4 plays a key role in the physiologic response of articular cartilage to loading via the generation of TRPV4-mediated intracellular Ca^2+^ transients in response to loading of tissue^18^,^19^,^20^. In humans, functional mutations of *TRPV4* have been linked to skeletal dysplasias and impaired joint health^21^,^22^,^23^. Furthermore, global deletion of *Trpv4* has been shown to accelerate OA progression in aging and obesity animal models of OA^24^,^25^,^26^. However, the role of TRPV4-mediated chondrocyte mechanotransduction in influencing OA disease pathogenesis specifically during the development and progression of the disease remains uncertain, as TRPV4 functions in other cell types within and around diarthrodial joints, including osteoclasts, synoviocytes, and adipocytes, innervating nerve fibers, as well as macrophages and other inflammatory cells^24^,^25^,^27^, all which could conceivably contribute to OA pathogenesis. Through the use of inducible and cartilage-specific transgenic mouse models, we have the ability to define the role of specific genes and pathways during disease progression in a temporal and tissue-specific fashion^28^.

The goal of this study was to test the hypothesis that TRPV4-mediated cartilage mechanotransduction plays a role in the pathogenesis of aging- and injury-induced OA. We first developed and validated an inducible, cartilage-specific *Trpv4* knockout (cKO) mouse model, which was then used to elucidate the role of TRPV4-mediated cartilage mechanotransduction during aging-associated or injury-induced OA.

## Results

### Animal Model Generation

*Col2a1-CreER*^*T2*^ and *Trpv4*^*lox*/*lox*^ mice on a C57BL/6 background were crossed and interbred for six generations. *Col2a1-CreER*^*T2*+^*;Trpv4*^*lox*/*lox*^ (cKO) and *Col2a1-CreER*^*T2*−/−^;*Trpv4*^*lox*/*lox*^ (WT) littermates were induced with parental tamoxifen at 10 weeks of age. cKO mice did not demonstrate any overt differences in feeding, behavior, or gait compared to WT mice, and the weights of the mice in both the aging and DMM studies did not differ from WT mice (Fig. S1).

### Functional knockout of TRPV4 in articular chondrocytes

**Figure 1:**
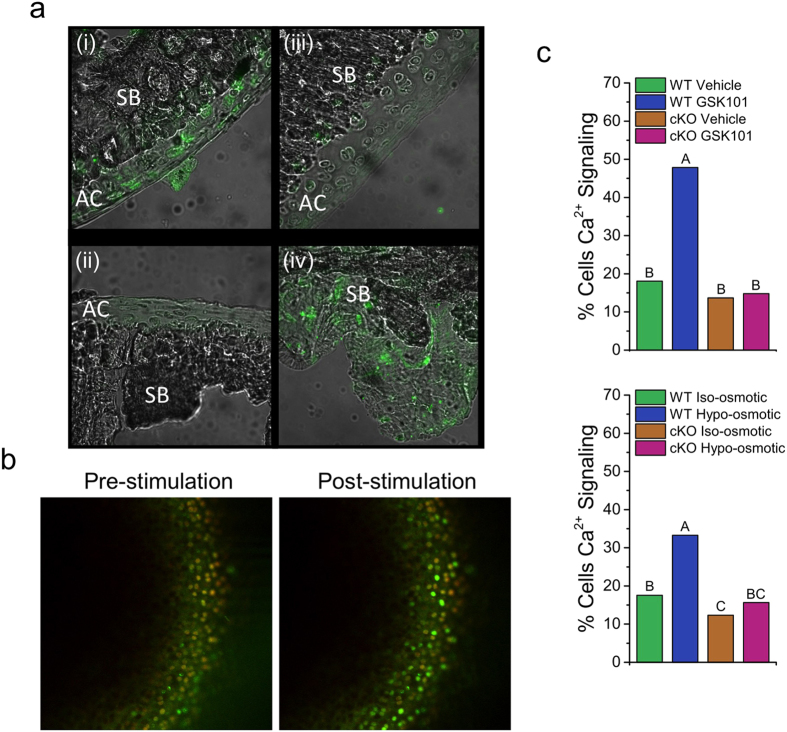
Inducible loss of TRPV4-mediated Ca^2+^ signaling in articular cartilage. (**a**) Immunofluorescence staining for TRPV4 two weeks following tamoxifen induction. Positive TRPV4 staining in WT mouse articular cartilage (i), absence of staining in the no primary control (ii) and cKO articular cartilage (iii), positive TRPV4 staining in cKO subchondral bone (iv). AC: articular cartilage; SB: Subchondral bone. (**b**) Representative confocal images of *in situ* chondrocyte intracellular Ca^2+^ signaling. (**c**) Percentage of chondrocytes responding to TRPV4 agonist GSK101 and hypo-osmotic stimulation. Ca^2+^ signaling in response to GSK101 and hypo-osmotic loading is present in WT chondrocytes and absent in cKO chondrocytes. Data not sharing a common superscript letter indicate a significant difference. p < 0.05, n = 3–9 (Bars do not have error bars because the percent responding metric does not have an error associated with it.).

Verification of TRPV4 knockout was performed two weeks after tamoxifen induction. Immunofluorescence demonstrated complete loss of TRPV4 in the articular cartilage of cKO mice, with TRPV4 staining persistent in the subchondral bone and marrow (Fig. 1). These findings confirm cartilage specificity of CRE expression, although other joint tissues such as tendon or synovium were not directly examined. However, it is important to note that with this system, knockout may also occur in other *Col2a1*-expressing tissues such as growth plate or intervertebral disc. Two weeks following tamoxifen induction, *in situ* WT chondrocytes exhibited baseline intracellular Ca^2+^ signaling in approximately 20% of cells in the absence of TRPV4 channel stimulation. In response to hypo-osmotic or GSK101 treatment, WT chondrocytes showed a significant two-fold increase in the percentage of chondrocytes exhibiting a Ca^2+^ signal (p < 0.05) (Fig. 1, Video S1 and Video S2). Chondrocytes from cKO mice maintained an equivalent level of basal signaling when compared to WT chondrocytes, but had no increase in signaling in response to GSK101 or hypo-osmotic stress, signifying a complete functional loss of TRPV4-mediated Ca^2+^ signal transduction.

### Loss of chondrocyte TRPV4 in adulthood reduces aging-associated OA severity

**Figure 2:**
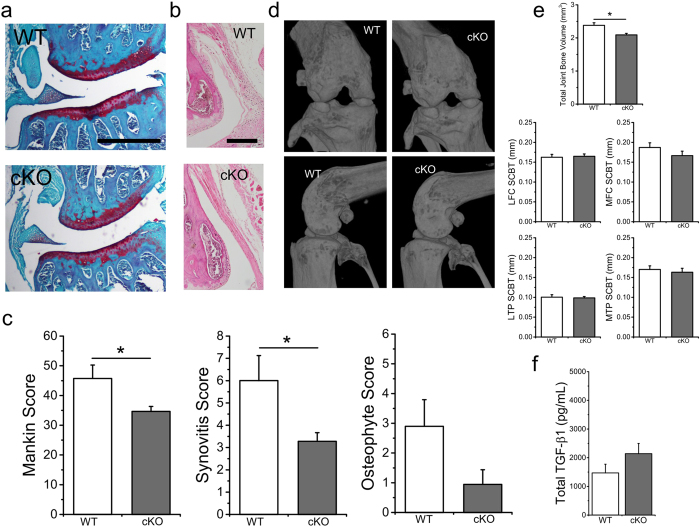
Loss of chondrocyte TRPV4 in adulthood reduced aging-associated OA severity and total joint bone volume with age. (**a**) Representative coronal sections of 1-year-old wild type (WT) and induced, cartilage-specific TRPV4 knockout (cKO) joints. (**b**) Representative histology of the joint synovial lining. (**c**) cKO mice exhibit significantly less OA severity, including Mankin and synovitis grading (p = 0.031 and p = 0.038, respectively). The difference in osteophyte scores is not significant (p = 0.153). (**d**) 3D microCT reconstructions show gross bony morphology of cKO joints is similar to that of WT mice. (**e**) cKO animals have significantly decreased total joint bone volume (p = 0.002). No significant differences in SCBT were seen between cKO and WT mice (MFC SCBT p = 0.158). (**f**) Total TGF-β1 levels in the synovial fluid of mouse hind limbs at sacrifice trends higher in cKO mice (p = 0.167). n = 12–13, mean + SEM. *p < 0.05.

Following induced TRPV4 knockout, WT and cKO mice were aged to 12 months. Several histological parameters were assessed to evaluate the severity and characteristics of osteoarthritic changes, including Modified Mankin grading, synovitis grading, and osteophyte grading (Fig. 2). cKO decreased OA severity as evaluated by Mankin grading (p = 0.031). Each component of the Mankin grading was also evaluated individually, revealing significantly lower Safranin-O staining in the WT mice than cKO (p = 0.029), as well as a trend for less structural degeneration in the cKO mice (p = 0.065), (Table 1). Synovitis was also found to be significantly lower in the cKO compared to WT mice (Fig. 2) (p = 0.038). No significant change in osteophyte scores in the cKO was observed (p = 0.153).

### Loss of chondrocyte TRPV4 is associated with decreased total periarticular bone volume

We examined multiple parameters of the periarticular bone, as OA is associated with increased subchondral bone density and remodeling. In contrast to the diffuse heterotopic ossification found in previous pan-TRPV4 knockout mouse models^24^, cKO animals had lower total joint bone volume (TJBV, p = 0.002) (Fig. 2). cKO mice also had increased bone mineral density (BMD) of the subchondral bone underlying the medial tibial plateau (p = 0.032) with no significant differences in thickness or volume of the subchondral bone underlying each joint quadrant (Fig. S2). We further investigated this relationship between cartilage TRPV4 signaling and the bone phenotype by measuring the level of total synovial fluid transforming growth factor (TGF)-β1 in the hind limbs immediately following sacrifice (Fig. 2), where we found a trend for higher TGF-β1 in the cKO mice (p = 0.167).

### Loss of chondrocyte TRPV4 in the setting of joint destabilization does not alter OA pathogenesis

**Figure 3:**
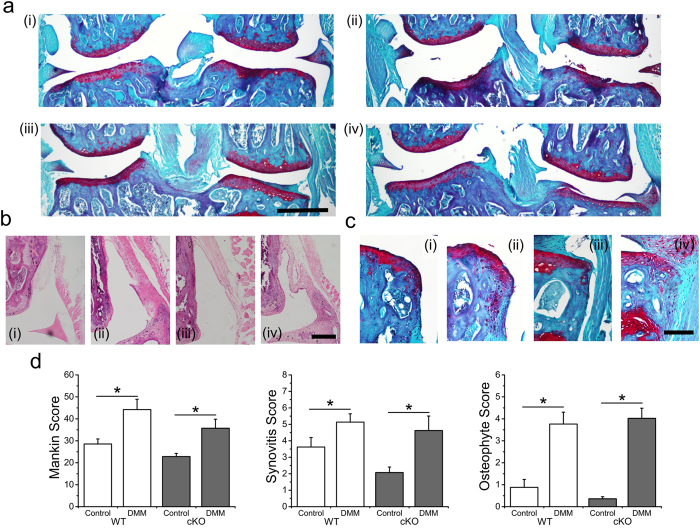
Loss of chondrocyte TRPV4 did not alter OA pathogenesis following joint destabilization. (**a**) Representative coronal sections of Control and DMM joints from wild type (WT) and induced, cartilage-specific TRPV4 knockout (cKO) joints. (i) WT Control, (ii) WT DMM, (iii) cKO Control, (iv) cKO DMM (**b**) Representative histology of the joint synovial lining (**c**) Representative histology of tibial plateau osteophytes (**d**) DMM causes significant osteoarthritic changes in both WT and cKO mice. (Control vs DMM comparisons: Mankin: WT p = 0.004, cKO p = 0.010; Synovitis: WT p = 0.005, cKO p = 0.023; Osteophyte: WT p = 0.001, cKO p < 0.001). n = 14–15, mean + SEM. *p < 0.05.

Unoperated limbs in both cKO and WT strains had a similarly mild degree of degenerative changes at 6 months of age, with no significant differences between genotypes with respect to Mankin scoring (p = 0.244), synovitis scoring (p = 0.226), or osteophyte scoring (p = 0.514) (Fig. 3, Table 2). Comparing control to DMM limbs, both cKO and WT mice developed similar degrees of post-traumatic OA as shown by increased Mankin scoring, synovitis, and osteophytes (Mankin: WT p = 0.004, cKO p = 0.010; Synovitis: WT p = 0.005, cKO p = 0.023; Osteophyte: WT p = 0.001, cKO p < 0.001). In both WT and cKO mice, DMM limbs exhibited increased cartilage structural degeneration and Safranin-O loss (Table 2).

### DMM produces a significant increase in total periarticular bone volume

**Figure 4:**
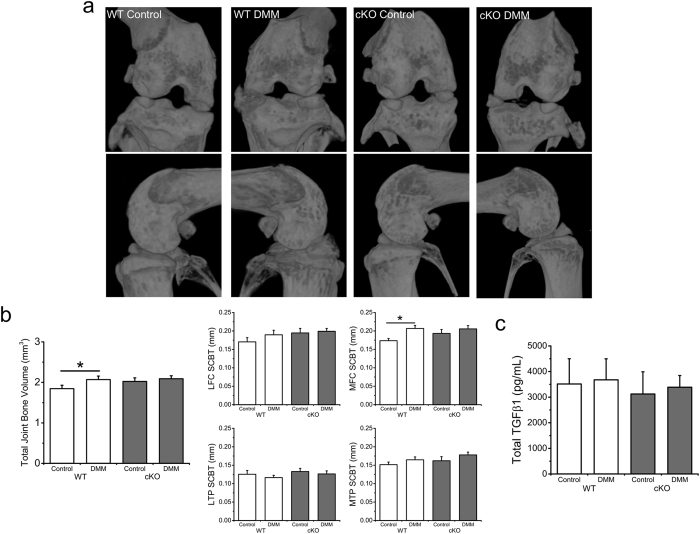
Loss of TRPV4 altered periarticular bone changes in the setting of joint destabilization. (**a**) 3D microCT reconstructions of wild type (WT) and induced, cartilage-specific TRPV4 knockout (cKO) joints. (**b**) DMM caused a significant increase in TJBV in WT mice (p = 0.049), an effect that was not significant in cKO mice (p = 0.198). DMM also caused a significant increase in MFC SCBT in WT animals (p = 0.002), not seen in cKO mice (p = 0.197). (**c**) Total TGF-β1 levels in the synovial fluid of mouse hind limbs at sacrifice. n = 14–15, mean + SEM. *p < 0.05.

Control limb TJBV did not differ across genotypes (p = 0.133) (Fig. 4). In WT mice, TJBV was significantly greater in DMM limbs compared to control limbs (p = 0.049), with no significant difference in the cKO mice (p = 0.198). With respect to the additional bone parameters measured, medial femoral condyle (MFC) subchondral bone thickness (SCBT) was greater in DMM than control limbs in WT mice (p = 0.002) but no significant difference was observed in cKO mice (p = 0.197, Fig. S2). Lateral femoral condyle (LFC) BMD was found to be increased in the cKO control limb compared to the WT control limb (p = 0.011). No differences were found in synovial fluid total TGF-β1 at the time of sacrifice (Fig. 4).

## Discussion

Cartilage homeostasis involves mechanically-regulated biologic processes, providing a potential opportunity for the discovery of disease-modifying OA drugs (DMOADS) to prevent aging-associated or post-traumatic OA development and progression by targeting cartilage mechanotransduction pathways^3^. Cartilage-specific, inducible transgenic animal models are a powerful tool for determining the role of specific signaling pathways during OA disease pathogenesis. In this study, we evaluated the effect of TRPV4-mediated cartilage mechanotransduction on aging-associated and injury-induced OA using an adult-induced, cartilage-specific TRPV4 knockout mouse. As measured by OA and synovitis scores, selective and induced loss of TRPV4 signaling in chondrocytes significantly decreased the severity of OA disease progression with aging, but did not alter injury-induced OA disease progression. These findings support the role of mechanobiologic factors during OA pathogenesis, with TRPV4 functioning as an important chondrocyte signaling molecule that facilitates development of OA with aging. These findings clearly render TRPV4-mediated calcium ion signaling a potential target for drug treatment to improve age-associated OA. Our findings also suggest that different OA subtypes, namely aging-associated versus post-injury, may be mediated through distinct biologic and mechanical mechanisms.

Previous studies using *Trpv4* pan-knockout animals showed that loss of TRPV4 signaling accelerated aging-associated OA development^24^,^25^. However, the recently-established roles of TRPV4 in periarticular tissues, including synovium^26^,^27^, bone^29^, muscle^30^, and adipose^31^, as well the known metabolic phenotype of animals with embryonic deletion of *Trpv4* 25,31, provided a clear rational to examine the role of TRPV4 specifically in adult articular cartilage.

The use of tissue-specific knockout or mutation of ion channels is an emerging field of study that provides methods for determining the potential mechanistic role and function of these important biological transducers^32^,^33^,^34^. Critical to these experiments is the verification of these genetic alterations *in vivo*. To achieve this, we cross-bred two well described and validated animal strains, one designed for inducible cartilage-specific Cre expression driven by the Col2a1 gene^28^ and the other for conditional *Trpv4* knockout by Cre-mediated excision^35^. We further elected to use two methodologies to validate this newly generated animal model of cartilage TRPV4 signaling. First, we used immunologic labeling of the TRPV4 protein, which strongly supported a reduced abundance of the ion channel specifically in the cartilage of the cKO. Next, we assessed the channel’s functional activity, as channel expression levels can often correlate poorly with channel signal transduction. To do this, we quantitatively analyzed *in situ* chondrocyte Ca^2+^ signaling to directly measure the activity of TRPV4 in the WT and cKO animals, finding complete insensitivity to two forms of TRPV4 channel stimulation in the cKO.

In contrast to pan-*Trpv4* knockout mice, which develop severe idiopathic OA with age, we find that loss of TRPV4 signaling in adult mouse cartilage alone attenuates aging-associated OA progression. A major phenotypic difference between the pan-knockout mice previously studied and the cartilage-specific knockout mice used in this study was the presence of significant bone formation and heterotopic ossification in the pan-knockouts. In this study, cartilage-specific deletion of *Trpv4* showed a decrease in total joint bone volume and a trend towards decreased osteophytes in the joint. The findings in this study support the hypothesis that the joint phenotype observed in the pan-knockouts is related to the role of TRPV4 in osteoclast function^29^,^36^,^37^, as the significant ectopic bone formed by the pan-knockouts, which could easily contribute to cartilage degeneration and OA, was entirely absent in the cartilage-specific knockouts. Cartilage-specific deletion of *Trpv4* in this study also showed a decreased level of joint synovitis. Modulation of soluble inflammatory mediators generated through chondrocyte TRPV4 signaling may be involved in the reduced synovitis in the cKO mouse, or this could simply represent a generally decreased level of intra-articular inflammation due to the reduced cartilage destruction.

In chondrocytes, the TRPV4 ion channel plays a critical role in the mechanical generation of intracellular Ca^2+^ signaling that regulates the anabolic and anti-catabolic response of the tissue to dynamic compressive loading^19^, although growing evidence shows that additional mechanotransduction elements such as the primary cilia and ATP signaling are also involved in this pathway^18^,^38^,^39^. The anabolic response to dynamic loading appears to be mediated by the upregulation of transforming growth factor (TGF)-β3 and potentially other members and modulators of the TGF-β family^40^. While TGF-β has a generally anabolic effect on chondrocytes^41^ and subchondral bone cells, recent studies have implicated this TGF-β-mediated anabolism and bone-cartilage crosstalk as an important regulator of age-related OA^42^,^43^. Furthermore, other studies have demonstrated increased osteophyte formation and OA severity following intra-articular TGF-β injection into the joint^44^. We sought to obtain *in vivo* evidence of TRPV4-mediated regulation of TGF-β signaling associated with the protective OA phenotype and reduced osteophytosis. Unfortunately, we are currently limited in our ability to thoroughly interrogate the TGF-β signaling pathway due to the small volume of synovial fluid in murine synovial joints. Future studies may wish to measure active, rather than total TGF-β1, as it is a more precise measure of TGF-β signaling, as well as other TGF-β signaling pathway modulators, such as TGF-β3 19 and follistatin^40^, two TGF-β-associated genes previously identified as TRPV4-responsive.

TRPV4 cKO did not prevent OA following joint instability caused by DMM. It is generally accepted that this type of post-traumatic OA is a more rapidly progressive and severe model of OA, and thus may be more difficult to modulate via genetic alterations. Our findings here and in previous studies support the notion that TRPV4 serves as a transducer of physiologic loading^19^, and that alternative “pathologic” mechanical signaling pathways can be activated following cartilage injury. For example, the PIEZO family of ion channels was recently identified as conferring mechanosensitivity of chondrocytes to high strains, also regulating chondrocyte injury response and cell death^45^. This would provide a possible explanation for why preventing TRPV4-mediated calcium ion signaling alone was not sufficient to prevent OA progression. The interaction between PIEZO and TRPV4 channels^45^ also suggests that multimodal therapeutic approaches may be necessary. Our findings also support the notion that the mechanisms involved in aging-related OA differ significantly from those in injury-induced OA.

The results of this study has led us to ask if inhibition of TRPV4 through pharmacologic means can similarly provide protection from age-related OA pathogenesis^46^. Indeed, the potential of therapeutically targeting TRPV4 is being actively investigated in several disease states^35^,^47^,^48^. A better understanding of the role of TRPV4 in the adjacent tissues, such as synovium, bone, and meniscus, particularly during OA pathogenesis, will likely also be important to this end. We also believe identifying disease-relevant downstream targets of chondrocyte TRPV4-mediated Ca^2+^ signaling^40^, in both young as well as aged and/or osteoarthritic cartilage, may provide additional, cartilage-specific, candidate targets for disease-modifying OA therapeutics. Interestingly, the contrasting role of TRPV4 in young^19^ and aged cartilage begets the question if epigenetic changes with aging are playing a pivotal role in the signaling that occurs downstream of cartilage mechanotransduction^49^,^50^. Lastly, given the vast channelome expressed by chondrocytes^51^,^52^, we predict that leveraging higher throughput approaches for evaluating the potential of candidate ion channels^19^,^53^,^54^,^55^ will be advantageous in identifying therapeutic targets for OA and other joint diseases.

## Methods

### Generation of transgenic mice

All procedures were performed in accordance with a protocol approved by the Duke University Institutional Animal Care and Use Committee. *Col2a1-CreER*^*T2*^ mice were crossed with *Trpv4*^*lox*/*lox*^ transgenic mice (used previously to generate epidermal-specific *Trpv4* null mice^35^). Genotyping was performed by Transnetyx (Cordova, TN). Male *Col2a1-CreER*^*T2*+^*;Trpv4*^*lox*/*lox*^ (cKO) and *Col2a1-CreER*^*T2*−/−^;*Trpv4*^*lox*/*lox*^ (WT) littermates were induced with tamoxifen (Sigma, 1 mg/10 g body weight, IP, daily for 3 days) at 10 weeks of age. Mice of both genotypes were randomly housed together in cages of 3–4 mice per cage. Mice were sacrificed for immunohistochemistry and functional Ca^2+^ imaging two weeks after induction.

### Immunolabeling

For immunolabeling, cKO and WT mouse knees were fixed in 4% paraformaldehyde overnight and cryosectioned (8 μm thick). Slides were blocked with 10% goat serum, incubated with the TRPV4 antibody (1:1000, ACC-034, Alomone, Jerusalem, Israel), followed by the Alexa Fluor 633 conjugated secondary (1:1000, A-21070, Life Technologies, Carlsbad, CA).

### Calcium Imaging

Chondrocyte TRPV4 channel function was evaluated by *in situ* fluorescence ratio imaging of Ca^2+^ indicator dyes, using previously described methods^24^. Briefly, freshly-dissected medial femoral condyles of 12 week old WT and cKO mice were imaged on a confocal microscope, and intracellular Ca^2+^ signaling was evaluated in response to the TRPV4 agonists GSK1016780A (GSK101, Sigma, 2 nM) and hypo-osmotic stress (−100 mOsm), (n = 3–4 condyles imaged per group).

### Destabilization of the Medical Meniscus (DMM) Surgery

Two weeks following tamoxifen induction at 10 weeks of age, 3 month old cKO and WT mice (n = 14–15) underwent destabilization of their left hind limb via transection of the medial meniscotibial ligament, as described previously^11^. Briefly, mice were induced under anesthesia via isofluorane induction and an incision was made on the anteriomedial knee. The patellar ligament was divided to access the infrapatellar fat pad and medial meniscal transverse ligament. Following visualization of the medial meniscus, its anterior insertion was transected. The joint capsule was sutured closed and tissue adhesive was applied to close the skin. Mice in the DMM study were sacrificed at 6 months of age, 3 months following DMM surgery. Aged cKO and WT mice were induced in an identical manner as described without any surgical intervention and sacrificed at 12 months of age (n = 12–13).

### MicroCT Imaging

Immediately following sacrifice, hind limbs were formalin-fixed and scanned in 70% ethanol (Skyscan 1176 MicroCT, Bruker, Billerica, MA). The following parameters were used for reconstruction: Dynamic range 0.0975 to 0.1025, Beam Hardening 40, Ring Artifact 6. A post-alignment parameter was set specifically for each image.

The following parameters were evaluated: Total joint bone volume (TJBV; a measure of all bone volume between the femoral and tibial growth plate, excluding calcified menisci), subchondral bone thickness (SCBT; calculated as the mean of three measurements taken from a sagittal section in the middle of each joint region), bone mineral density (BMD), and bone volume (BV). SCBT, BMD and BV were calculated separately for four joint regions: the medial and lateral femoral condyles (MFC & LFC) and medial and lateral tibial plateaus (MTP & LTP). BMD and BV were calculated for the trabecular regions of each region, delineated proximally by the first subchondral axial slice with trabecular bone to one slice prior to the growth plate.

### Histology

Following scanning, knee joints were decalcified (Cal-Ex Decalcification Solution; Fisher Scientific), dehydrated, embedded in paraffin via an automated tissue processor (ASP300S; Leica Microsystems), and sectioned using a microtome (8 μm coronal slices). Sections were stained with Hematoxylin, Fast Green, and Safranin-O for semi-quantitative modified Mankin and osteophyte scoring, or Hematoxylin and Eosin for synovitis grading as previously described^56^. Briefly, digital micrographs were taken of the slides and the scores from three blinded graders for individual joint quadrants (medial femoral condyle, medial tibial plateau, lateral femoral condyle, and lateral tibial plateau) as well as for the whole joint, were averaged.

### Synovial Fluid Biomarker Assays

Synovial fluid was collected at sacrifice for biomarker analysis^57^. Total TGF-β1 was measured using Sandwich ELISA (Legend Max #436707, BioLegend, San Diego, CA, USA). Samples were run undiluted as recommended by the manufacturer. For the purposes of statistical analyses, in each of the assays, ½ the lower limit of detection (LLOD), which is considered the minimum detectable dose, was substituted for any value that was determined to be below the limit of detection. The minimum detectable concentration of total TGF-β1 was reported as 3.5 pg/mL. The intra-assay coefficient of variability was 3.2%.

### Statistical Analysis

Statistics were performed with JMP Pro 12 (SAS, Cary, NC). Nominal Ca^2+^ signaling data were compared between treatment groups using the chi-square test. Student’s *t* test (for normally distributed data) and Wilcoxon Sign Rank test (for non-normally distributed data) was used for single comparisons of body composition and aging data (normally distributed: body weight, TJBV, SCBT, BV, BMD, total TGF-β; non-normally distributed: total Mankin scoring). A matched pairs *t* test (for normally distributed data) and matched pairs Wilcoxon Sign Rank test (for non-normally distributed data) was used to report significance between the contralateral Control and operated DMM limbs, with ANOVA (or Kruskal-Wallis for non-normally distributed data) with Bonferroni correction used to compare across genotypes (normally distributed: body weight, body fat, TJBV, SCBT, BV, BMD, Mankin scoring, total TGF-β; non-normally distributed: synovitis scoring, osteophyte scoring). Significance was indicated at p < 0.05.

## Additional Information

**How to cite this article**: O’Conor, C. J. *et al*. Cartilage-Specific Knockout of the Mechanosensory Ion Channel TRPV4 Decreases Age-Related Osteoarthritis. *Sci. Rep*. **6**, 29053; doi: 10.1038/srep29053 (2016).

## Supplementary Material

Supplementary Information

Supplementary Video S1

Supplementary Video S2

## Figures and Tables

**Table 1:** Mankin scoring of aging joint histology by individual component.

Parameter	WT	cKO	Genotype (p-value)
Cartilage Degeneration	18.8 ± 2.7	13.0 ± 1.0	0.065
Safranin-O Loss	19.6 ± 1.6	15.3 ± 0.9*	**0**.**029**
Tidemark Duplication	0.89 ± 0.49	0.28 ± 0.17	0.375
Chondrocyte Cloning	0.26 ± 0.11	0.36 ± 0.12	0.347
Hypertrophic Chondrocytes	2.2 ± 0.3	2.3 ± 0.3	0.908
Fibrocartilage	0	0	–
Subchondral BoneThickness	4.0 ± 0.3	3.5 ± 0.2	0.207

Statistical differences between WT and cKO were determined by Student’s *t* test for normally distributed data (cartilage degeneration, Safranin-O loss, hypertrophic chondrocytes, and subchondral bone thickness) or Wilcoxon Sign Rank test for non-normally distributed data (tidemark duplication, chondrocyte cloning). Mean ± SEM. *p < 0.05.

**Table 2:** Mankin scoring of DMM joint histology by individual component.

Parameter	WT			cKO		
	Control	DMM	Limb(p-value)	Control	DMM	Limb(p-value)
Cartilage Degeneration	9.9 ± 1.1	18.6 ± 2.6*	**0**.**007**	7.5 ± 0.8	13.8 ± 2.3*	**0**.**0419**
Safranin-O Loss	12.4 ± 1.2	19.5 ± 2.2*	**0**.**0113**	10.2 ± 0.1	15.8 ± 1.9*	**0**.**0083**
Tidemark Duplication	0.24 ± 0.12	0.87 ± 0.41	0.375	0.071 ± 0.71	0.071 ± 0.71	1
Chondrocyte cloning	0.31 ± .08	0.26 ± 0.09	0.8438	0.38 ± 0.12	0.33 ± 0.10	0.7646
Hypertrophic Chondrocytes	1.75 ± 0.45	1.80 ± 0.56	0.6975	1.97 ± 0.47	2.07 ± 0.48	0.751
Fibrocartilage	0	0	–	0	0.07 ± 0.05	0.5
Subchondral Bone Thickness	3.91 ± 0.46	3.38 ± 0.43	0.4801	2.62 ± 0.32	3.55 ± 0.42	0.124

Statistical differences between Control and DMM limbs were determined by a matched pairs *t* test for normally distributed data (Safranin-O loss) or Wilcoxon Sign Rank test for non-normally distributed data (cartilage degeneration, tidemark duplication, chondrocyte cloning, hypertrophic chondrocytes, fibrocartilage, and subchondral bone thickness). No genotype effects were found. Mean ± SEM. *p < 0.05.
