# Supplementary material for: Cartilage-Specific Knockout of the Mechanosensory Ion Channel TRPV4 Decreases Age-Related Osteoarthritis
Source: Sci Rep. 2016 Jul 8;6:29053. doi: 10.1038/srep29053 (PMC4937413; doi:10.1038/srep29053)
Supplement: Supplementary Information [file srep29053-s1.pdf]

SUPPLEMENTARY INFORMATION

Cartilage-Specific Knockout of the Mechanosensory Ion Channel TRPV4 Decreases Age-Related Osteoarthritis

Christopher J. O'Conor[a,b,c], Sendhilnathan Ramalingam[c], Nicole A. Zelenski[c], Halei C. Benefield[b,c], Isaura Rigo[c], Dianne Little[c], Chia-Lung Wu[f,g], Di Chen[d], Wolfgang Liedtke[e], Amy L. McNulty[c], Farshid Guilak[f,g,b,*]

[a]Department of Pathology & Immunology, Washington University in St. Louis, Missouri, 63110
[b]UNC/NCSU Joint Department of Biomedical Engineering, UNC School of Medicine, Chapel Hill, NC 27599
[c]Department of Orthopaedic Surgery, Duke University Medical Center, Durham NC 27710
[d]Department of Biochemistry, Rush University, Chicago, IL
[e]Department of Neurology and Neurobiology, Duke University Medical Center, Durham NC 27710
[f]Department of Orthopaedic Surgery, Washington University in St. Louis, Missouri, 63110
[g]Shriners Hospitals for Children – St. Louis, St. Louis, Missouri 63110

Corresponding author:
Farshid Guilak, Ph.D.
Department of Orthopaedic Surgery
Washington University and Shriners Hospitals for Children – St. Louis Hospital
3210 McKinley Research Building
St. Louis, Missouri 63110
Email: guilak@wustl.edu

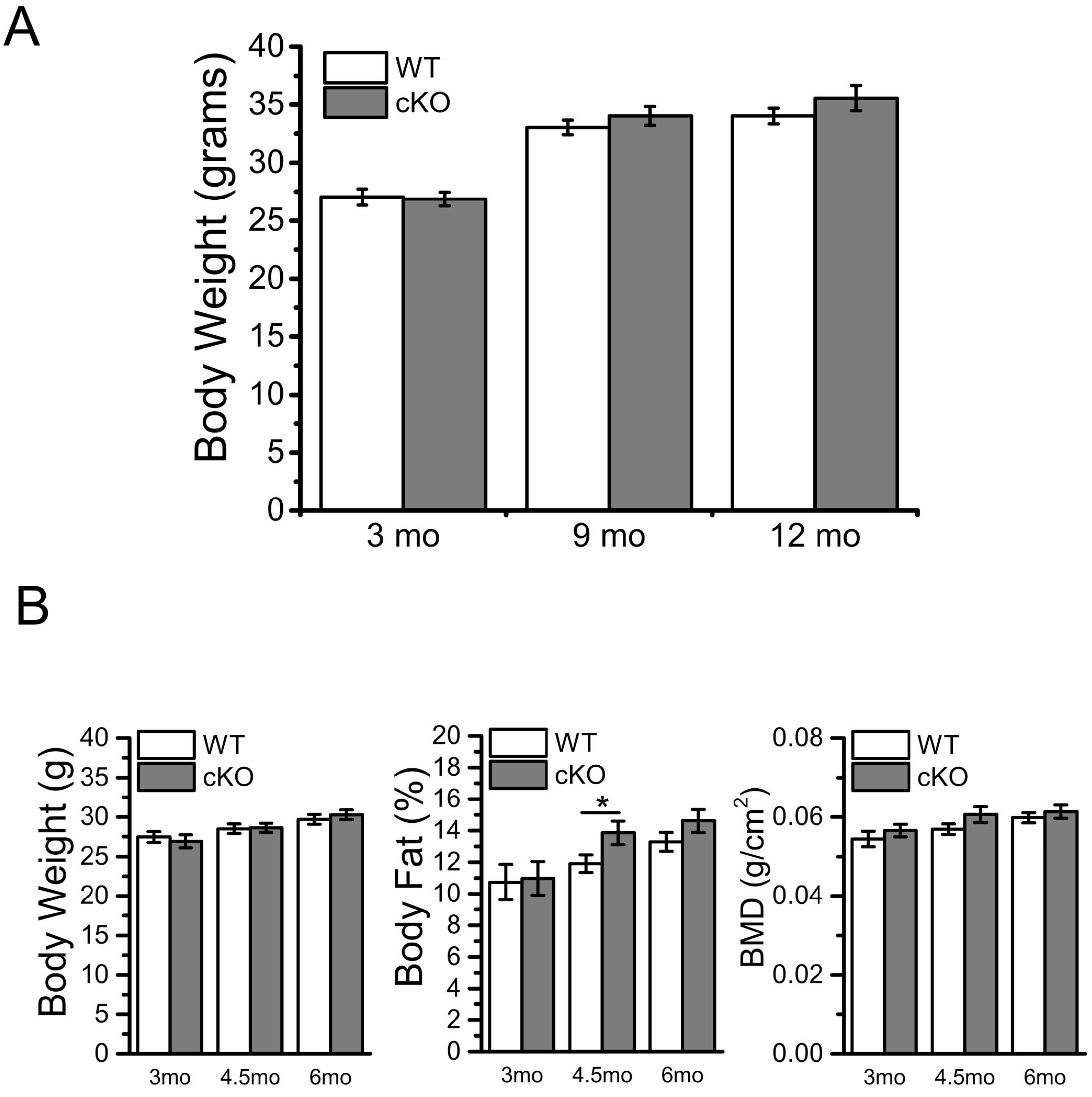

Fig. S1. Body composition of WT and cKO animals. **(A)** Body weights with aging. **(B)** Body weight, total body fat, and total body bone mineral density of mice that underwent DMM at 3 months of age. cKO had significantly higher body fat percentage at 4.5 months old ($p=0.045$). Mean±SEM. *$p<0.05$.

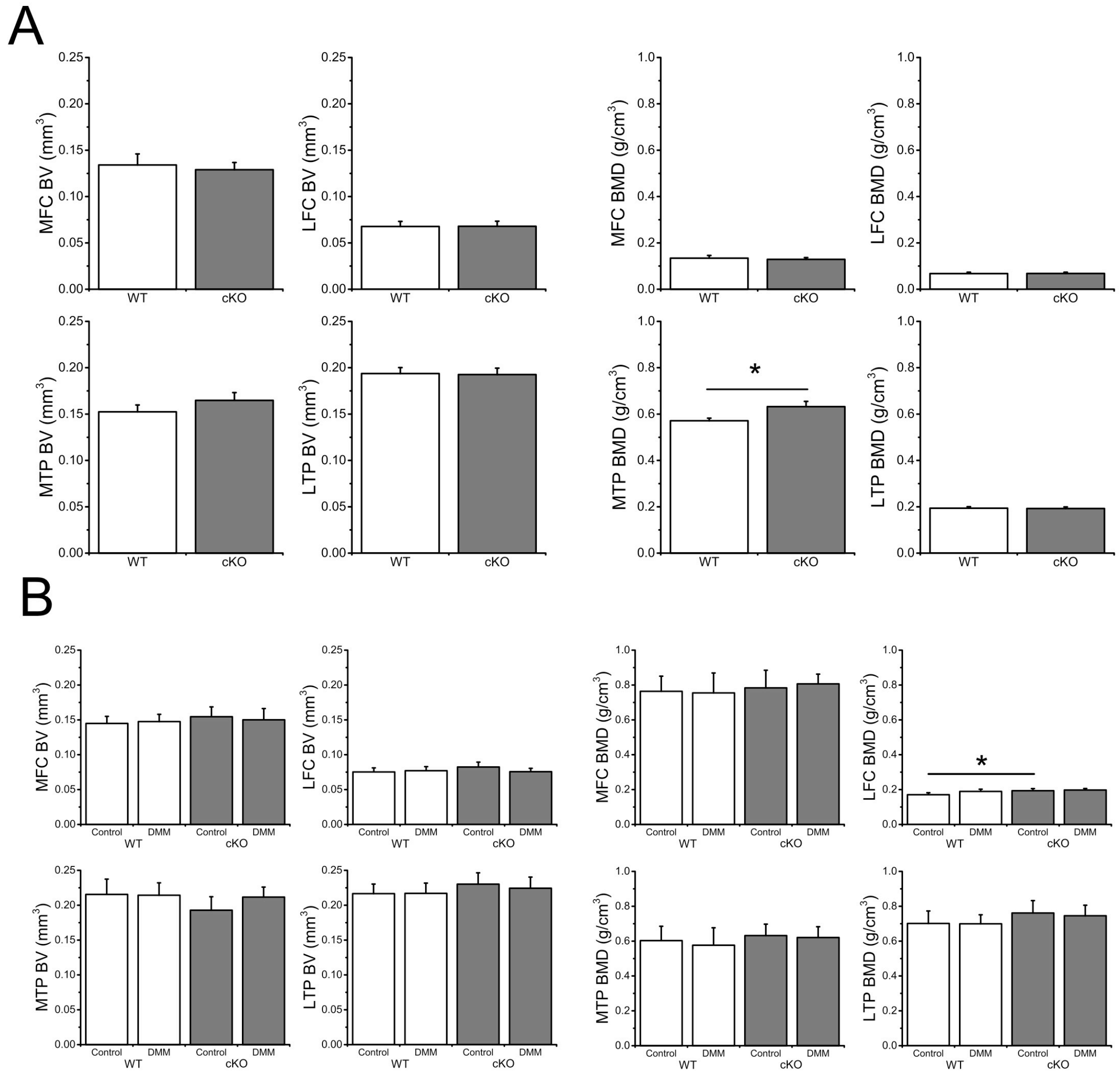

Fig. S2. microCT analysis of bone volume (BV) and bone mineral density (BMD) in each joint quadrant. **(A)** Aged cKO mice have increased BMD of the subchondral bone underlying the medial tibial plateau (p=0.032). **(B)** BMD of the lateral femoral condyle was increased in the WT Control limb compared to KO control limb (p=0.011). Mean+SEM. *p <0.05.

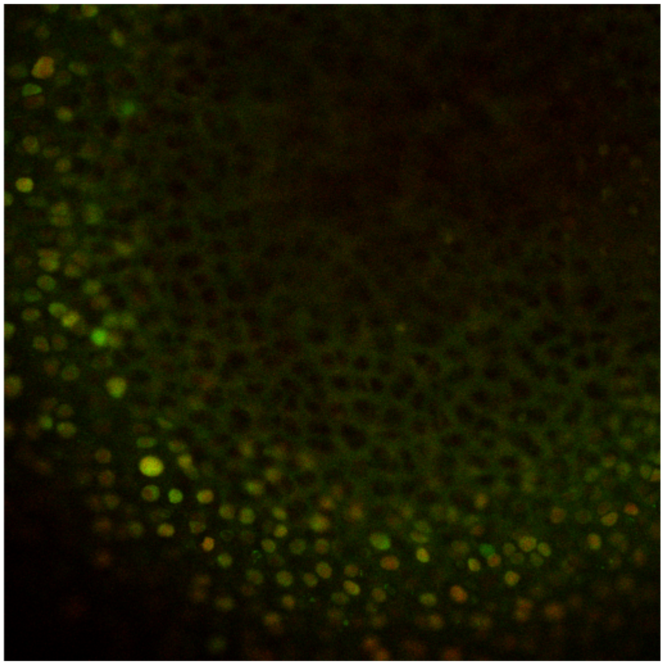

Video S1. *In situ* intracellular Ca^{2+} signaling in freshly dissected mouse articular cartilage. This video depicts the response of chondrocytes in wild type mice to the TRPV4 agonist GSK101.

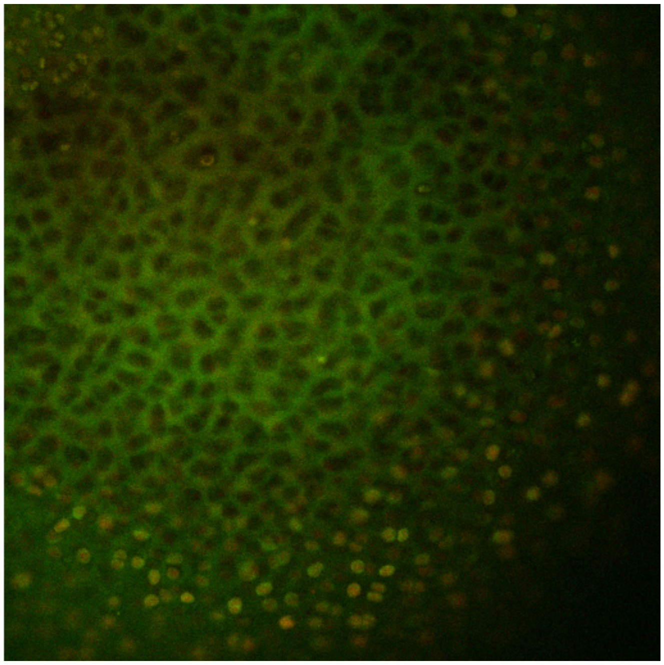

Video S2. *In situ* intracellular Ca^{2+} signaling in freshly dissected mouse articular cartilage. This video depicts the response of chondrocytes in *Trpv4* cKO mice to the TRPV4 agonist GSK101.
